# Optimization, Validation and Application of HPLC-PDA Methods for Quantification of Triterpenoids in *Vaccinium vitis-idaea* L.

**DOI:** 10.3390/molecules26061645

**Published:** 2021-03-16

**Authors:** Gabriele Vilkickyte, Lina Raudone

**Affiliations:** 1 Laboratory of Biopharmaceutical Research, Institute of Pharmaceutical Technologies, Lithuanian University of Health Sciences, Sukileliu av. 13, LT-50162 Kaunas, Lithuania; lina.raudone@lsmuni.lt; 2 Department of Pharmacognosy, Lithuanian University of Health Sciences, Sukileliu av. 13, LT-50162 Kaunas, Lithuania

**Keywords:** triterpenoids, HPLC, validation, *Vaccinium vitis-idaea* L.

## Abstract

Triterpenoids have regained much attention as promising multi-targeting bioactive agents of natural origin in the treatment of numerous disorders. Due to the high potential for phytopharmaceutical development, accurate qualitative and quantitative analysis of triterpenoids for screening and quality control is required. *Vaccinium vitis-idaea* L. (lingonberry) raw materials have aroused interest as a rich source of triterpenoids. However, currently, no validated, rapid, and easy-to-perform quantification method is available for the routine control of these compounds in lingonberries. This research aimed at developing and validating HPLC-PDA methods for the determination and screening of triterpenoids in extracts of lingonberry leaves, fruits, and flowers. The developed methods were deemed satisfactory by validation, which revealed acceptable analytical specificity, linearity (*r^2^* > 0.9999), precision (RSD < 2%), trueness (94.70–105.81%), and sensitivity (LOD: 0.08–0.65 µg/mL). The real sample analysis demonstrated established methods applicability for quantification of 13 triterpenoids in lingonberries and emphasized differences between raw materials. Lingonberry fruits were distinguished by the richness of ursolic acid; lingonberry flowers by similar profile to fruits, but low content of neutral triterpenoids; whereas lingonberry leaves by the particularly high level of α-amyrin. Thus, the proposed methods proved to be reliable and applicable for quantification and routine analysis of triterpenoids in lingonberry samples.

## 1. Introduction

One of the most important classes of secondary plant metabolites and bioactive compounds is triterpenoids [1]. These compounds are distinguished by their high structural, functional diversity, and potential medicinal application in numerous disorders [2,3,4]. It has been reported about more than 50,000 triterpenoids derived from the acyclic hydrocarbon, squalene. Pentacyclic triterpenoids can be divided into main classes of oleanane (oleonolic, maslinic acids, erythrodiol, β-amyrin), ursane (ursolic acid, uvaol, α-amyrin), lupane (betulinic acid, betulin, lupeol), or friedelane (friedelin) series triterpenoids, based on their parent skeleton [5,6,7,8]. More complex triterpenoids include phytosterols with a predominant member of β-sitosterol [9]. Triterpenoids have been shown from various scientific studies to have a wide range of beneficial bioactivities, namely, chemopreventive, anti-inflammatory, antiviral, antihyperlipidemic, antidiabetic, hepatoprotective, antibacterial, antioxidant, and wound-healing properties [8,10,11]. Therefore, they constitute a source of interest as promising leading compounds for the development of new multi-targeting pharmaceuticals [5,8].

Characterization of triterpenoids can be carried out by a variety of chromatographic techniques, but the simultaneous determination of triterpenoids is rather challenging considering their similar structures and polarities, as well as the limitations of methodologies [7,11,12]. The use of conventional procedures such as thin-layer chromatography (TLC) for the identification of triterpenoids may be deceptive and precision or accuracy are considered to be lower compared to other methods [13,14]. Commonly used gas chromatography (GC) methods offer better separation efficiency, however, due to the low volatility and high molecular weight of triterpenoids, the derivatization step prior to analysis is required. That makes the analysis laborious and long [6,15,16,17,18]. In this sense, high-performance liquid chromatography (HPLC) methods have gained more interest as the method of choice for the analysis of naturally non-volatile compounds as triterpenoids. Considering the detection systems of triterpenoids, some drawbacks have also been found. Because neutral triterpenoids—betulin, erythrodiol, lupeol, as well as phytosterols, have just a few polar functional groups and possess high lipophilicity, they cannot be easily ionized and be detected by mass spectrometry (MS); also, there is the possibility of ion suppression effects in complex matrices and a relatively narrow dynamic range [13,19,20]. Some detection systems, such as nuclear magnetic resonance (NMR), are quite difficult to use due to the complexity of triterpenoids present in plants [21]. The widest application was found by reversed-phase HPLC with ultraviolet (UV) or photo diode array (PDA) detection, as non-selective and universal detectors. The key problem with this technique is that most triterpenoids due to their structural peculiarities lack chromophores and have very low UV absorption [11,15,20,21]. In order to get better sensitivity, detection at low wavelengths 205–210 nm against intense solvent absorption is needed, leading to the limited and demanding choice of the mobile phase and other chromatographic parameters [6,7,12,15,17]. Despite its shortcomings, the HPLC-PDA method has significant benefits in terms of simplicity, versatility, reliability, reproducible performance, and relatively low costs [21,22,23].

The presence of triterpenoids has been reported in many medicinal plants, widespread in various parts such as fruit, leaves, stems, or roots. As the component of edible plants, triterpenoids are naturally included in the human diet [5,6,11,21]. Due to pharmacological relevance, the intensive investigations of natural sources of triterpenoids continue to be very important. Although there is much evidence about triterpenoids occurrence in plants, their quantitative distribution has rarely been studied directly [5,18]. Among all plants studied so far, members of the *Ericaceae* family are distinguished by the richness and particular diversity of triterpenoids [24]. *Vaccinium vitis-idaea* L. (lingonberry), especially leaves of this plant, can be regarded to be one of the least studied raw materials in the *Ericaceae* family in terms of triterpenoids, because most studies have tended to focus on phenolic constituents [25,26,27]. This evergreen subshrub with edible fruits has been used for a long time in traditional medicine for the treatment of urinary tract disorders, gastric, liver, skin, respiratory, and rheumatic diseases, because of its strong antioxidant, antiseptic, anti-inflammatory, anti-cough, diuretic, reparative, and anticancer activities [26,27,28,29]. Since these multi-biological activities are associated with the phytochemical composition and prevailing phytochemical markers, the distribution of triterpenoids in lingonberry raw materials renders to be an important quality trait for quality and authenticity control.

In the light of previous reports, only a few studies have been conducted on the evaluation of lingonberry triterpenoids composition. Most studies in this field were carried out on non-validated GC-MS/FID methods with long analyses duration and derivatization or other sample preparation and time-consuming steps [24,30,31,32]. High waste in terms of reagents together with the multi-step prolonged analysis may be a limiting factor for routine analysis. Other studies have only focused on main triterpenoid acids [33,34]. As far as we know, currently, no validated, rapid, and easy-to-perform quantification method is available for the routine control of different groups of triterpenoids in lingonberries.

In this paper, we report findings of two fully validated, cost-effective, rapid, and accurate quantifications of triterpenoids HPLC-PDA methods, which are shown to be applicable for lingonberry raw materials. To the best of our knowledge, characterization of triterpenoids in lingonberry flowers has never been reported so far, also this is the first comparative report describing different predominant triterpenoids in leaves, fruits, and flowers of lingonberries.

## 2. Results and Discussion

### 2.1. Optimization of Methods

In this study, the chromatographic performance of two columns—ACE C18 (150 × 4.6 mm, 3 μm) and ACE C18 (250 × 4.6 mm, 5 μm) was evaluated. Column type had no significant effect on peak shape and resolution of the main chromatographic peaks, but a column ACE C18 (150 × 4.6 mm, 3 μm) was preferred because of the notably reduced time of the chromatographic run. Since detection of triterpenoids with weak chromophores is highly dependent on the mobile phase [12], consideration of mobile phase composition was the key factor of optimization. On account of reports on triterpenoids analysis [11,12,14,35,36], different compositions of the mobile phase, namely, acetonitrile:water, methanol:water, methanol:acetonitrile, acetonitrile:tetrahydrofuran:methanol:water, acetonitrile:tetrahydrofuran, acetonitrile:water:acetic acid, methanol:water:acetic acid, acetonitrile:methanol:water, were investigated in various proportions and in gradient and isocratic elution patterns. Retention times of most triterpenoids decreased as the amount of organic solvent in the proportion of mobile phase increased. However, with one chromatographic mode, it was not possible to detect all tested triterpenoids. The mobile phase consisting of acetonitrile and water (89:11, *v/v*) was found to be suitable for analysis of most triterpenoids with chromophores (maslinic, corosolic, betulinic, oleanolic, ursolic acids, betulin, erythrodiol, uvaol), whereas phytosterol (β-sitosterol) or neutral triterpenoids, which lack chromophores (lupeol, β-amyrin, α-amyrin, friedelin), were detected only with a mobile phase, composed of acetonitrile and methanol (10:90, *v/v*). In line with the results of Gleńsk and Włodarczyk [23], methanol superiority versus acetonitrile in terms of detection of triterpenoids and a more stable baseline at low wavelength could be pointed out. Gradient pattern way of two combined methods was rejected due to big changes in backpressure during analysis, very long chromatographic run time, and other different chromatographic conditions, required for acceptable separation of different triterpenoids. An isocratic elution requires only one pump and minimizes the variation of baseline and ghost peaks [37]. Therefore, two simple isocratic mode methods for different triterpenoids were further optimized.

The column temperature is considered an important tool in optimizing parameters of system suitability and separation quality [19]. We have performed the analysis at different temperatures ranging from 20–35 °C in order to improve resolution and selectivity of triterpenoids, mainly the separation between oleanolic and ursolic acids and between β-sitosterol and α-amyrin. Although an increase in temperature decreased chromatographic run time and shortened retention times of oleanolic and ursolic acids, but also reduced resolution between these peaks (Supplementary Figure S1). Finally, a column temperature of 20 °C was selected as a final condition for the detection of triterpenoids with chromophores. On the contrary, a column temperature of 35 °C was employed for detection of triterpenoids, which lack chromophores, because the decrease in temperature led to hindering of peaks resolution and co-elution of β-sitosterol and α-amyrin (Supplementary Figure S2). The flow rate was modified accordingly to temperature changes in the range of 0.5–1 mL/min. As high temperature reduces viscosity and thus pressure [38], a higher flow rate at higher temperatures could be selected. Bearing this factor in mind, acceptable results were obtained with a flow rate of 0.7 mL/min for detection of triterpenoids with chromophores at a column temperature of 20 °C and 1 mL/min for detection of triterpenoids, which lack chromophores, at a column temperature of 35 °C. Injection volumes of 10 µL were found to produce adequate detector responses (tested range 10–20 µL), thus confirming the sensitivity of both methods.

As described above, chromatographic conditions provided acceptable separation of various triterpenoids with symmetrical peaks, reasonable resolution, and selectivity, with mean values of 4.0 and 1.2, respectively, for neighboring peaks. All compounds were successfully eluted within a relatively short time—16 min.

### 2.2. Validation of Methods

#### 2.2.1. Analytical Specificity

**Figure 1:**
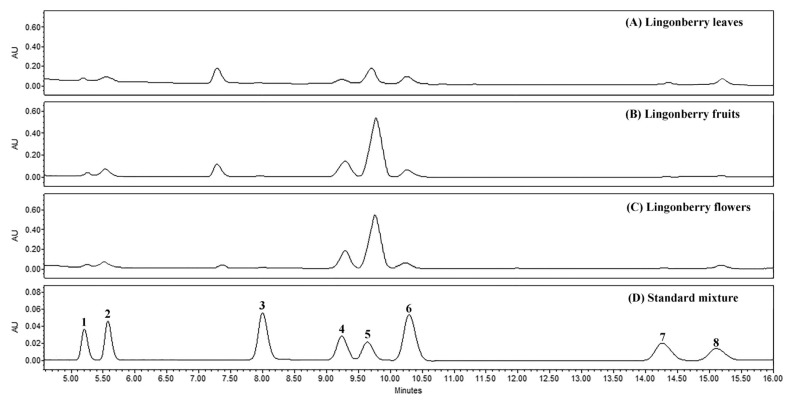
Representative HPLC-PDA chromatograms (λ = 205 nm) of extracts of lingonberry (**A**) leaves, (**B**) fruits, (**C**) flowers, and (**D**) standard mixture, showing the separation of triterpenoid acids and neutral triterpenoids with chromophores. Peak assignments: 1—maslinic acid, 2—corosolic acid, 3—betulinic acid, 4—oleanolic acid, 5—ursolic acid, 6—betulin, 7—erythrodiol, 8—uvaol.

**Figure 2:**
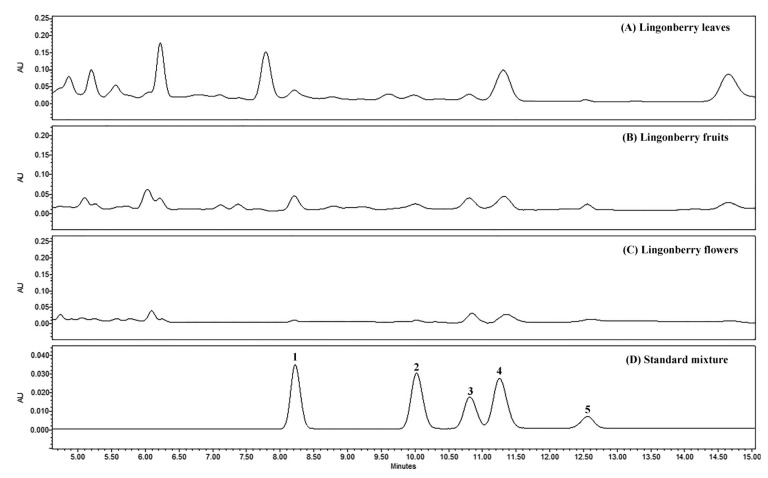
Representative HPLC-PDA chromatograms (λ = 205 nm) of extracts of lingonberry (**A**) leaves, (**B**) fruits, (**C**) flowers, and (**D**) standard mixture, showing the separation of phytosterol and neutral triterpenoids, which lack chromophores. Peak assignments: 1—lupeol, 2—β-amyrin, 3—β-sitosterol, 4—α-amyrin, 5—friedelin.

The blank solutions, standard mixtures of triterpenoids, and lingonberry samples were analyzed under the conditions of optimized methods. The specificity of peaks in lingonberry samples was identified by comparing their retention times and UV spectra of analytes with those of standard compounds (Figure 1 and Figure 2). It was found for all samples that there were no interferences at the regions of retention times of interest. Well-resolved peaks indicated reasonable analytical specificity of methods and the ability to distinguish analytes from other lingonberry matrix components.

#### 2.2.2. Linearity, Working Range and Limits

Calibration curves of all triterpenoids showed excellent linear response, obtaining determination coefficients (*r*^2^) greater than 0.9999 over the working range 0.26–800.00 μg/mL, as shown in Table 1. Linearity coefficients >0.9999 are generally considered as evidence of acceptable fit of the data to the regression line [39]. Minimum concentration levels at which triterpenoids can be reliably detected (LOD) and quantified (LOQ) were in the range of 0.08–0.65 μg/mL and 0.24–1.78 μg/mL, respectively. Our obtained detection and quantification limits were lower than reported in several previous HPLC-PDA/UV studies of triterpenoids, demonstrating the sensitivity of our analysis. We report approx. three times lower LOD and LOQ values of lupeol and ursolic acid, four times lower values of oleanolic acid, and even more than five times lower values of maslinic, corosolic acids, and β-sitosterol, compared to other researchers [12,40,41].

#### 2.2.3. Precision

Precision (distribution of data values) is considered as one of the most important elements of the chromatographic test, which can be affected by numerous factors, such as reproducibility of injections, analyst skills, sample preparation, failure to control chromatographic condition, and other aspects [42]. The data of intra-day repeatability, intermediate precision, and total repeatability of our proposed analysis of triterpenoids are represented in Table 2. The analysis was found to be precise with intra-day and inter-day variability (% RSD) for peak areas of all tested triterpenoids in the range of 0.28–0.80% and 0.32–1.05%, respectively, which are well within generally accepted prescribed limits (<2%) [43]. The total repeatability of 18 analytes (0.35–1.09%) did not exceed the proposed acceptable values, thus indicating highly reproducible results.

#### 2.2.4. Trueness

The trueness of the method at concentrations around the critical values of the working range allows the evaluation of random and systematic errors of the qualitative results and shows agreement between measured and real values [44]. Guidelines propose different acceptance criteria with acceptable recoveries in a range of at least 80–120% for our studied concentration levels [45,46]. As shown in Table 3, the trueness of measurements was within the acceptable range, allowing the accurate analysis of various triterpenoids. The percentage recoveries of tested triterpenoids were 94.70–104.74%, 100.19–105.81%, and 99.39–101.63% at low, medium, and high concentrations of range, respectively, with the mean value of trueness—101.49% and RSD—0.59%.

### 2.3. Application to Lingonberry Materials

The optimized HPLC-PDA methods were used to estimate the qualitative and quantitative composition of triterpenoids in extracts of leaves, fruits, and flowers of lingonberries. Thirteen compounds were assigned as triterpenoids, belonging to subgroups of triterpenoid acids, neutral triterpenoids, and phytosterols (Table 4).

The profile of triterpenoid acids composed of maslinic, corosolic, betulinic, oleanolic, and ursolic acids. The sum of these acids was found to be higher in fruits and flowers (9541.29 and 9461.94 µg/g dry weight (DW), respectively) than in leaves, in which approx. four and a half times lower level of triterpenoid acids was estimated. Ursolic acid, followed by oleanolic acid was the main triterpenoid acid in all raw materials of lingonberry and the main constituent of fruits and flowers, accounting for 59% and 70%, respectively, of total identified triterpenoids (Supplementary Figure S3). The ratio of oleanolic and ursolic acid, as one of the possible characteristic factors of authenticity control, was found 1:4.6, 1:5.3, 1:4.9 for lingonberry leaves, fruits, and flowers, accordingly. The significant prevalence of ursolic acid, which has up to five times higher content compared to oleanolic acid, is consistent with what has been found in previous studies of lingonberry raw materials [24,33,34]. Since ursolic acid from *Vaccinium* fruits was determined to inhibit tumor cell growth, invasion, and metastasis [25], this compound can be considered as a promising anticancer agent of lingonberries.

The accumulation pattern of identified neutral triterpenoids, namely, betulin, erythrodiol, uvaol, lupeol, β-amyrin, α-amyrin, and friedelin in lingonberry raw materials appeared to be different. The highest contribution of them to total identified compounds was found in lingonberry leaves—63%, with a much lower contribution in other raw materials—fruits (22%) and flowers (9%). Lingonberry leaves distinguished by the significantly highest content of α-amyrin, accounted for 29% of total identified triterpenoids. The superiority in contents of α-amyrin in lingonberry leaves compared to fruits was directly in line with previous findings [31], showing that α-amyrin can be considered as a principal neutral triterpenoid of lingonberry leaves. Recently, α-amyrin and its derivatives were reported to display profound anti-inflammatory [47] and antimicrobial activity [48], thus indicating that these generally known biological properties of lingonberry leaves may be partially attributed to α-amyrin, as prevailing triterpenoids.

The second predominant neutral triterpenoid of lingonberry leaves was betulin, followed by other triterpenoids in this manner: lupeol > uvaol > friedelin > β-amyrin > erythrodiol. Although lingonberry fruits are distinguished by approx. three times lower content of α-amyrin and much lower levels of uvaol and erythrodiol, a similar tendency of predominant and minor neutral triterpenoids was obtained. Meanwhile, lingonberry flowers were characterized by principal neutral triterpenoid—betulin, accounting for 57% of total identified neutral triterpenoids with others occurring in significantly lower levels by this manner: α-amyrin > uvaol > β-amyrin > friedelin > erythrodiol > lupeol. Besides neutral triterpenoids, reported in the present study, fernenol, taraxasterol, ursenal, adriaticol, and various derivatives of friedelin were found in lingonberries earlier [31,32].

The current analysis of phytosterols, chemically called steroids, found in lingonberries, led to the identification of the principal component—β-sitosterol. This compound constituted about 7% of the total identified triterpenoids in all tested materials. Contrary to the findings of Szakiel et al. [31], which reported that phytosterol fraction in lingonberry leaves was almost 4-fold larger than in fruits, our study showed the 1.7-fold higher content of β-sitosterol in lingonberry fruits, compared to leaves. This can be justified in part by different geographical origin and extraction method of lingonberry raw materials. Moreover, the phytosterol profile of lingonberries is composed not only of β-sitosterol but also campesterol, cycloartanol, 24-methylenecycloartanol, sitostanol, stigmasterol, tremulone, lanosterol, stigmastanol, and cycloartenyl acetate can be detected [24,27]. However the sum of other phytosterols was reported to be significantly lower than the content of the main compound—β-sitosterol, reaching up to 83% and 93% of total identified steroids in lingonberry fruits and leaves, respectively [31]. Lingonberry raw materials seem to be a good source of β-sitosterol, which still holds great potential as future therapeutics, particularly because of its cholesterol-lowering properties [49].

Summarizing, the total identified content of triterpenoids was greatest in lingonberry fruits (13,397.88 µg/g DW), followed by flowers (11,180.06 µg/g DW) and leaves (7020.25 µg/g DW). The sum of identified triterpenoids in lingonberry fruits and leaves was partly in line with the previous study, bearing in mind that the level of triterpenoids highly depends on geographical origin, harvesting time, and stage of maturity of raw material [31], thus driving the potential of further investigations. It was pointed out that triterpenoids are often more predominant in fruits than in leaves [24]. Some authors have compared the composition of triterpenoids in lingonberry leaves and fruits with other *Vaccinium* members and have drawn conclusions that lingonberry triterpenoids profile is rather similar to a cranberry, blueberry, and bilberry, but with considerably greater quantities of most components [30,32,50]. Vrancheva et al. reported that lingonberry leaves predominantly contained relative concentrations of phytosterols and almost twice the content of other triterpenoids compared to other *Vaccinium* species tested [27]. This highlights the lingonberry richness of multi-targeting bioactive agents—triterpenoids. Regarding triterpenoids profile of lingonberry raw materials, key findings of our present study emerge: lingonberry fruits can be characterized by their richness of triterpenoid acids, mainly ursolic acid; lingonberry flowers—by similar profile to fruits, but much lower levels of neutral triterpenoids; whereas lingonberry leaves by the particularly high content of α-amyrin.

## 3. Materials and Methods

### 3.1. Chemicals and Solvents

Analytical and chromatographic grade chemicals and solvents were used for this study: acetonitrile, methanol, tetrahydrofuran, acetic acid, α-amyrin, β-amyrin, β-sitosterol, lupeol, erythrodiol, maslinic and oleanolic acids from Sigma-Aldrich (Steinheim, Germany); uvaol, friedelin, betulin, betulinic and corosolic acids from Extrasynthese (Genay, France); and ursolic acid from Carl Roth (Karlsruhe, Germany). Ultrapure water was supplied by a Milli-Q water purification system from Millipore (Bedford, MA, USA).

### 3.2. Standard Solutions

Stock solutions of all triterpenoids were prepared in methanol at 200 µg/mL and stored at −20 °C until use. A serial dilution was made on each stock solution with methanol to prepare working standard solutions at concentrations in the linear range. Combined working solutions of standards of triterpenoids were obtained by the mixing stock solutions.

### 3.3. Plant Material

Fresh and fully grown lingonberry raw materials were collected from 10 clonal plants at the natural growing site in North-East Lithuania (altitude 110 m; 56°00′40.6′′ N 25°31′29.4′′ E) in June 2020 (in the case of flowers) and September 2020 (in the case of fruits and leaves). Flowers and leaves were air-dried at room temperature, and fruits were freeze-dried at 0.01 mbar pressure and −85 °C condenser temperature in Zirbus sublimator (Zirbus Technology GmbH, Bad Grund, Germany). Fully-dried raw materials were ground with a Retsch 200 mill (Haan, Germany) to obtain a fine powder, and dark storage in sealed containers until extraction.

### 3.4. Preparation of Lingonberry Extracts

Extraction conditions were selected and further modified on the basis of previous researches [36,40]. Each sample, comprising of 1 g of powdered lingonberry flowers, fruits, or leaves, was immersed in 10 mL methanol and subjected to ultrasound-assisted extraction for 25 min in an Elmasonic P ultrasonic bath (Singen, Germany) at room temperature. The extractive solutions were then centrifuged for 30 min at 3000× *g* in a Biofuge Stratos centrifuge. Extraction was performed in triplicate (*n* = 3) for each raw material. Before the injection into the HPLC system, the extracts were filtered by using 0.22-μm pore size membrane filters (Carl Roth GmbH, Karlsruhe, Germany).

### 3.5. Optimization and Validation of Chromatographic Analysis

The chromatographic conditions for analytical methods were optimized and validated using standard mixtures of triterpenoids, and suitability was checked on matrixes of lingonberry raw materials. The effect of column type and mobile phase composition on the chromatographic behavior were evaluated. The chromatographic optimization included factors like injection volume, the temperature of the column, and the flow rate of the mobile phase as well. Optimization was based on the peak resolution, selectivity, and retention time.

The proposed analytical methods were validated by following the International Council for Harmonisation (ICH) guidelines and taking into account the recommendations and validation criteria of EURACHEM and EU guidelines [43,45,46]. The following criteria were analyzed: analytical specificity, the linearity of detector response, the limit of detection (LOD), the limit of quantification (LOQ), working range, precision, trueness, system suitability, and method applicability.

The method’s specificity was verified by the ability to detect triterpenoids of interest in samples of lingonberries by comparing and overlaying visible chromatograms of stand-ards with those found in samples. To verify the interference of the matrix constituents, blank analyses were also performed by injecting mobile phases. For the quantitation of triterpenoids, at least five-point calibration curves were generated by linear regression analysis, as a function of peak areas (y) versus concentrations (x) of each standard. Linearity was determined by calculating coefficients of determination (*r*^2^) of obtained regression lines. The validation study included the range around the critical values of calibration curves. The LOD and LOQ were determined at a signal-to-noise (S/N) ratio of 3 and 10, respectively. To test precision, six replicates of analytes on the same day (*n* = 6, for intra-day repeatability) were injected and the same procedure was repeated on three consecutive days (*n* = 3, for inter-day intermediate precision) with total repeatability of 18 replicates (*n* = 18). The precision was determined from percentage relative standard deviations (% RSD) of peak areas. The acceptable percent of total repeatability was calculated according to the Horwitz equation, as an exponential relationship between RSD and a dimensionless mass fraction (C): % RSD = 2^(1−0.5logC)^ [45]. To check the trueness, experiments using the standard addition method were performed by spiking the known amount of standards with a blank matrix at three different levels (low, medium, and high concentration of a particular compound linearity range). Triplicate analysis (*n* = 3) of the study at each level was performed and results were expressed as percentage recoveries of spiked triterpenoids. Values of resolution and selectivity were calculated by the System Suitability option in Empower (Waters) Software to verify if system suitability is acceptable for the analysis.

### 3.6. Chromatographic Analysis

The optimized chromatographic analysis was carried out using a Waters Alliance 2695 liquid chromatography system (Waters, Milford, CT, USA) equipped with 2996 photodiode array detector (Waters, Milford, CT, USA) and ACE C18 (150 × 4.6 mm, 3 μm) column (ACT, Aberdeen, UK). Since all triterpenoids cannot be separated in a single chromatographic run, different chromatographic conditions were used.

For the analysis of triterpenoid acids (maslinic, corosolic, betulinic, oleanolic, ursolic acids) and neutral triterpenoids with chromophores (betulin, erythrodiol, uvaol), the mobile phase consisted of acetonitrile and water (89:11, *v/v*), delivered at a flow rate of 0.7 mL/min in the isocratic mode. The column temperature was set at 20 °C with an injection volume of 10 μL. Whereas the isocratic elution system for the analysis of neutral triterpenoids, which lacks chromophores (lupeol, β-amyrin, α-amyrin, friedelin) and phytosterol (β-sitosterol), consisted of acetonitrile and methanol (10:90, *v/v*). The column temperature was set at 35 °C, the flow rate was 1 mL/min, and the sample injection volume was 10 μL.

Detection of all triterpenoids was performed at a wavelength of 205 nm corresponding to the maximum absorption, and peaks were identified with retention times as compared with standards.

### 3.7. Statistical Analysis

Statistical analysis was performed by IBM SPSS 26.0 (SPSS Inc., Chicago, IL, USA) and Microsoft Office Excel 2017 (Microsoft, Redmond, WA, USA). The quantitative results were expressed as means ± standard deviations (SD) from three replicates (*n* = 3) for each sample. The linear regression was used to calculate determination coefficients (*r*^2^) of the regression lines for each quantified triterpenoid. Obtained means between qualitative values were subjected to analysis by analysis of variance (ANOVA) followed by post hoc Tukey’s multiple comparison test. The accepted level of statistical significance was *p* < 0.05.

## 4. Conclusions

The HPLC-PDA quantification of triterpenoids methods developed in this work showed adequate validation parameters—analytical specificity, linearity, precision, and trueness, and limits of detection and quantification on μg/mL scale also allowed the characterization of 13 bioactive triterpenoids in extracts of lingonberry leaves, fruits, and flowers and brought up phytochemical markers for quality control. The preliminary experiments showed that lingonberry fruits and flowers could be an exploitable source of triterpenoid acids and phytosterols, whereas lingonberry leaves could be an exploitable source of neutral triterpenoids, such as α-amyrin. It could be concluded that the proposed methods are reliable, simple, cost-effective, and can be successfully applied for further triterpenoid routine analysis of lingonberries or related plant species.

## Figures and Tables

**Table 1:** Linearity and sensitivity data of triterpenoids.

Compound	Linear Equation	Coefficient of Determination (*r*^2^)	Linearity Range (µg/mL)	LOD (µg/mL)	LOQ (µg/mL)
Maslinic acid	y = 8960 x + 2060	0.99995	0.26–66.67	0.08	0.24
Corosolic acid	y = 6910 x + 1270	0.99991	0.26–66.67	0.16	0.48
Betulinic acid	y = 8970 x + 4310	0.99996	0.33–83.33	0.11	0.32
Oleanolic acid	y = 12,600 x + 8710	0.99994	1.56–200.00	0.21	0.65
Ursolic acid	y = 9040 x + 30,900	0.99998	3.13–800.00	0.26	0.82
Betulin	y = 10,600 x + 4350	0.99999	0.33–83.33	0.29	0.89
Erythrodiol	y = 12,800 x + 7740	0.99995	0.26–66.67	0.17	0.51
Uvaol	y = 9310 x + 4390	0.99993	0.26–66.67	0.30	0.99
Lupeol	y = 6740 x + 4740	0.99997	0.78–100.00	0.14	0.41
β-Amyrin	y = 7870 x + 4310	0.99999	0.78–100.00	0.14	0.43
β-Sitosterol	y = 3980 x + 3610	0.99992	0.78–100.00	0.37	1.13
α-Amyrin	y = 6470 x + 9440	0.99999	1.56–200.00	0.24	0.73
Friedelin	y = 1320 x + 5390	0.99991	1.56–100.00	0.65	1.78

**Table 2:** Precision data of triterpenoids.

Compound	Precision (% RSD)		Total Repeatability (% RSD, *n* = 18)	Proposed Acceptable Total Repeatability (% RSD)
	Intra-Day (*n* = 6)	Inter-Day (*n* = 3)		
Maslinic acid	0.47	0.68	0.78	1.17
Corosolic acid	0.54	1.05	1.06	1.28
Betulinic acid	0.42	0.45	0.67	1.33
Oleanolic acid	0.46	0.98	1.01	1.15
Ursolic acid	0.68	0.66	0.79	1.14
Betulin	0.44	0.96	0.75	1.33
Erythrodiol	0.49	0.43	0.86	1.16
Uvaol	0.58	0.69	1.00	1.17
Lupeol	0.28	0.32	0.35	1.26
β-Amyrin	0.67	0.63	0.54	1.24
β-Sitosterol	0.32	0.39	0.63	1.27
α-Amyrin	0.47	0.90	0.72	1.27
Friedelin	0.80	0.81	1.09	1.23

**Table 3:** Results of the recovery study of triterpenoids.

Compound	Low Concentration of Range		Medium Concentration of Range		High Concentration of Range	
	% Recovery	% RSD	% Recovery	% RSD	% Recovery	% RSD
Maslinic acid	94.70	0.22	103.89	0.17	99.84	0.12
Corosolic acid	100.72	0.83	105.26	1.04	99.39	0.35
Betulinic acid	102.23	0.96	100.19	0.26	99.65	0.16
Oleanolic acid	100.93	0.76	101.52	0.71	100.65	0.13
Ursolic acid	104.48	0.65	100.99	0.86	98.64	0.66
Betulin	102.92	0.63	100.50	0.70	99.88	0.23
Erythrodiol	104.74	1.07	103.35	0.24	99.95	0.09
Uvaol	95.96	1.12	104.39	1.03	100.09	0.08
Lupeol	102.78	0.88	105.81	0.25	99.96	0.12
β-Amyrin	103.62	0.57	103.84	1.20	100.03	0.07
β-Sitosterol	99.30	0.73	104.87	1.00	99.48	0.28
α-Amyrin	98.88	0.76	104.10	0.82	99.39	0.37
Friedelin	104.61	1.16	104.93	1.07	101.63	0.53

**Table 4:** Contents of triterpenoids (µg/g DW) in extracts of lingonberry leaves, fruits, and flowers.

Compound	Lingonberry Leaves	Lingonberry Fruits	Lingonberry Flowers
Maslinic acid	37.26 ± 0.78 ^a^	39.78 ± 0.88 ^a,b^	18.71 ± 0.39 ^a^
Corosolic acid	95.92 ± 2.97 ^a^	63.50 ± 1.71 ^a,b^	43.74 ± 1.01 ^a^
Betulinic acid	NQ	17.94 ± 0.41 ^a,b^	NQ
Oleanolic acid	351.07 ± 10.74 ^b^	1498.16 ± 45.13 ^c^	1607.48 ± 40.11 ^b^
Ursolic acid	1627.60 ± 60.33 ^c^	7921.91 ± 299.58 ^d^	7792.01 ± 256.13 ^c^
Sum of triterpenoid acids	2111.85	9541.29	9461.94
Betulin	756.24 ± 20.42 ^d^	753.67 ± 16.58 ^e,f^	546.89 ± 15.86 ^d^
Erythrodiol	87.61 ± 2.80 ^a^	6.36 ± 0.08 ^a^	27.49 ± 0.99 ^a^
Uvaol	328.54 ± 11.17 ^b^	45.08 ± 0.95 ^a,b^	75.81 ± 2.20 ^a^
Lupeol	638.28 ± 18.51 ^e^	547.69 ± 15.34 ^e^	17.08 ± 0.56 ^a^
β-Amyrin	220.38 ± 7.27 ^f^	232.17 ± 4.88 ^b^	49.71 ± 1.34 ^a^
α-Amyrin	2052.25 ± 79.18 ^i^	770.42 ± 20.13 ^e,f^	201.57 ± 4.51 ^f^
Friedelin	302.28 ± 9.37 ^b,f^	592.13 ± 13.03 ^e^	37.89 ± 1.01 ^a^
Sum of neutral triterpenoids	4385.58	2947.52	956.44
β-Sitosterol	522.82 ± 15.68 ^g^	909.07 ± 31.82 ^f^	761.68 ± 25.90 ^e^
Total identified	7020.25	13,397.88	11,180.06

Different letters within the same column indicate statistically significant (*p* < 0.05) differences between contents of identified compounds (values with no common letters are significantly different). NQ—not quantified (amount below LOQ).

## Data Availability

All data generated during this study are included in this article.

## Supplementary Materials

Figure S1: HPLC-PDA chromatogram (λ = 205 nm) of the standard mixture, showing the separation of triterpenoid acids and neutral triterpenoids with chromophores, at a column temperature of 30 °C. Peak assignments: 1—maslinic acid, 2—corosolic acid, 3—betulinic acid, 4—oleanolic acid, 5—ursolic acid, 6—betulin, 7—erythrodiol, 8—uvaol; Figure S2: HPLC-PDA chromatogram (λ = 205 nm) of the standard mixture, showing the separation of phytosterol and neutral triterpenoids, which lack chromophores, at a column temperature of 25 °C. Peak assignments: 1—lupeol, 2—β-amyrin, 3—β-sitosterol, 4—α-amyrin, 5—friedelin; Figure S3: Content of identified triterpenoids (%, W/DW) in extracts of lingonberry leaves (**A**), fruits (**B**), and flowers (**C**).

## Sample Availability

Samples of the compounds are available from the authors.
