# Supplementary material for: Optimization, Validation and Application of HPLC-PDA Methods for Quantification of Triterpenoids in Vaccinium vitis-idaea L
Source: Molecules. 2021 Mar 16;26(6):1645. doi: 10.3390/molecules26061645 (PMC8001753; doi:10.3390/molecules26061645)
Supplement: Supplementary file 1 [file molecules-26-01645-s001.pdf]

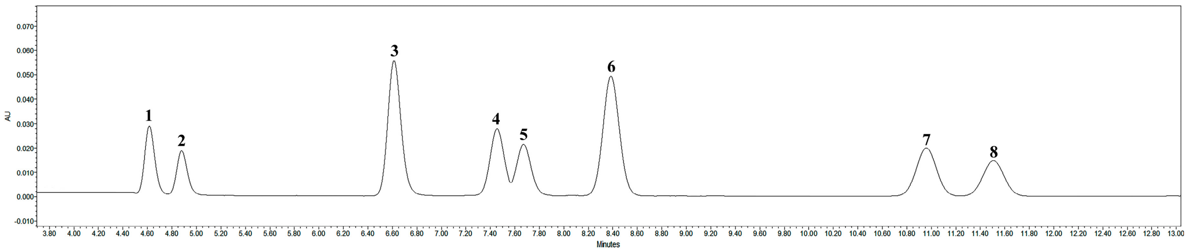

Figure S1. HPLC-PDA chromatogram (λ = 205 nm) of the standard mixture, showing the separation of triterpenoid acids and neutral triterpenoids with chromophores, at a column temperature of 30 °C. Peak assignments: 1—maslinic acid, 2—corosolic acid, 3—betulinic acid, 4—oleanolic acid, 5—ursolic acid, 6—betulin, 7—erythrodiol, 8—uvaol.

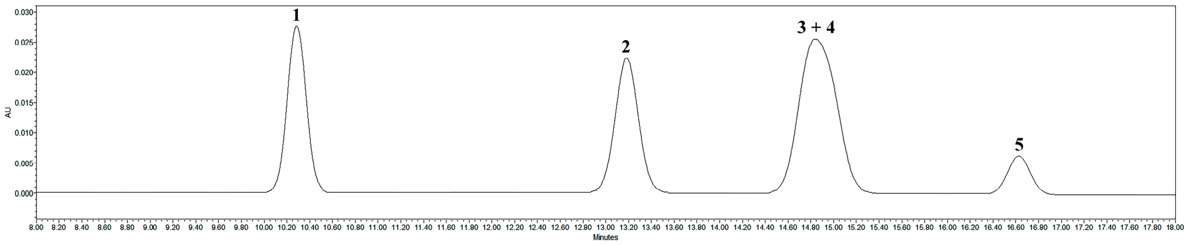

Figure S2. HPLC-PDA chromatogram (λ = 205 nm) of the standard mixture, showing the separation of phytosterol and neutral triterpenoids, which lack chromophores, at a column temperature of 25 °C. Peak assignments: 1—lupeol, 2—β-amyrin, 3—β-sitosterol, 4—α-amyrin, 5—friedelin.

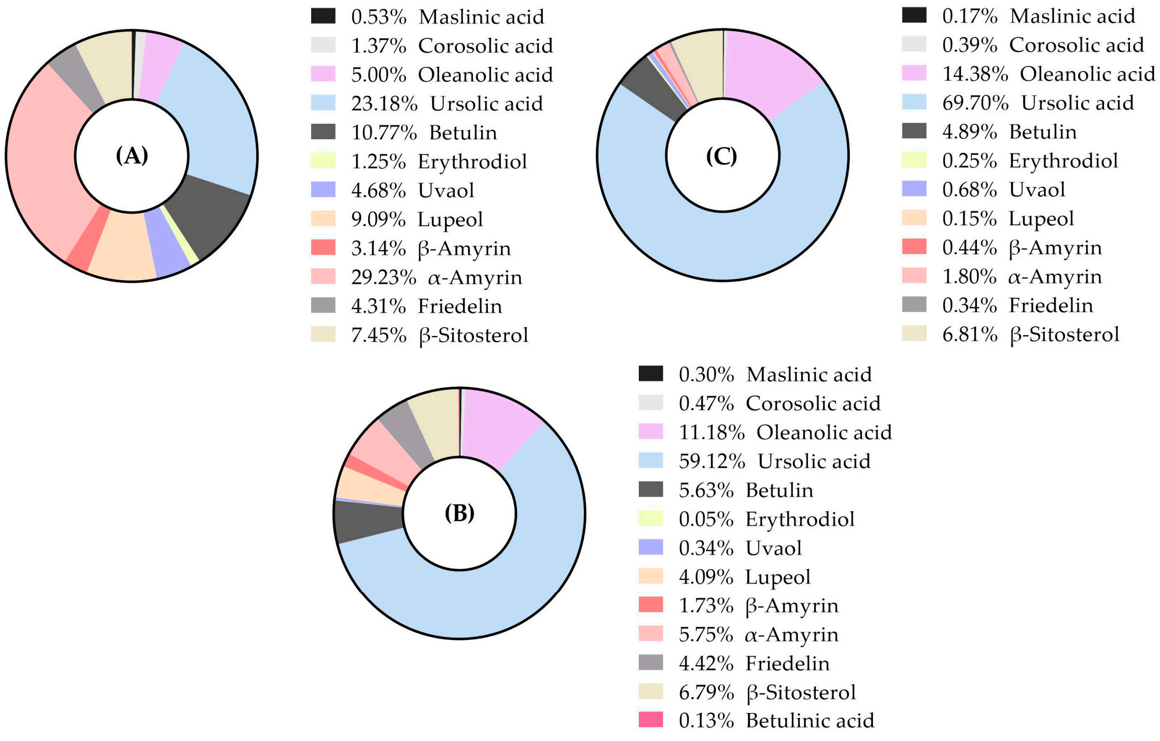

Figure S3. Content of identified triterpenoids (%, W/DW) in extracts of lingonberry leaves (**A**), fruits (**B**), and flowers (**C**).
